# Supplementary material for: Comprehensive identification of somatic nucleotide variants in human brain tissue
Source: Genome Biol. 2021 Mar 29;22:92. doi: 10.1186/s13059-021-02285-3 (PMC8006362; doi:10.1186/s13059-021-02285-3)
Supplement: Supplementary file 5 — Additional file 5. BSMN_Membership_List. [file 13059_2021_2285_MOESM5_ESM.pdf]

Name		Institution	E-mail	Comments
**Sara**	**Bizzotto**	Boston Children's Hospital	Sara.Bizzotto@CHILDRENS.HARVARD.EDU	
**Michael**	**Coulter**	Boston Children's Hospital	Michael_Coulter@HMS.HARVARD.EDU	
**Caroline**	**Dias**	Boston Children's Hospital	Caroline.Dias@CHILDRENS.HARVARD.EDU	
**Alissa**	**D'Gama**	Boston Children's Hospital	Alissa_D'Gama@HMS.HARVARD.EDU	
**Javier**	**Ganz**	Boston Children's Hospital	javier.ganz@CHILDRENS.HARVARD.EDU	
**Robert**	**Hill**	Boston Children's Hospital	rshill.wl@GMAIL.COM	
**August Yue**	**Huang**	Boston Children's Hospital	Yue.Huang@CHILDRENS.HARVARD.EDU	
**Sattar**	**Khoshkhoo**	Boston Children's Hospital	Sattar.Khoshkhoo@CHILDRENS.HARVARD.EDU	
**Sonia**	**Kim**	Boston Children's Hospital	soniakim@G.HARVARD.EDU	
**Alice**	**Lee**	Boston Children's Hospital	ejalice.lee@GMAIL.COM	
**Michael**	**Lodato**	Boston Children's Hospital	mlodato@GMAIL.COM	
**Eduardo**	**Maury**	Boston Children's Hospital	eduardo_maury@hms.harvard.edu	
**Michael**	**Miller**	Boston Children's Hospital	mbmiller@bwh.harvard.edu	
**Rebeca**	**Borges-Monroy**	Boston Children's Hospital	rborgesmonroy@G.HARVARD.EDU	
**Rachel**	**Rodin**	Boston Children's Hospital	rachelereiff@GMAIL.COM	
**Christopher A.**	**Walsh**	Boston Children's Hospital	Christopher.Walsh@CHILDRENS.HARVARD.EDU	
**Zinan**	**Zhou**	Boston Children's Hospital	Zinan.Zhou@CHILDRENS.HARVARD.EDU	
**Craig**	**Bohrson**	Harvard University	craigbohrson@GMAIL.COM	
**Chong**	**Chu**	Harvard University	chong_chu@HMS.HARVARD.EDU	
**Isidro**	**Cortes-Soriano**	Harvard University	isidrolauscher@GMAIL.COM	
**Yanmei**	**Dou**	Harvard University	Yanmei_Dou@HMS.HARVARD.EDU	
**Alon**	**Galor**	Harvard University	alon_galor@HMS.HARVARD.EDU	
**Doga**	**Gulhan**	Harvard University	Doga_Gulhan@HMS.HARVARD.EDU	
**Minseok**	**Kwon**	Harvard University	minseok_kwon@HMS.HARVARD.EDU	
**Joe**	**Luquette**	Harvard University	luquette@G.HARVARD.EDU	
**Peter**	**Park**	Harvard University	peter_park@HMS.HARVARD.EDU	
**Maxwell**	**Sherman**	Harvard University	Maxwell_Sherman@HMS.HARVARD.EDU	
**Vinay**	**Viswanadham**	Harvard University	vvv776@G.HARVARD.EDU	
**Schahram**	**Akbarian**	Icahn School of Medicine at Mount Sinai	schahram.akbarian@MSSM.EDU	
**Andrew**	**Chess**	Icahn School of Medicine at Mount Sinai	andrew.chess@MSSM.EDU	
**Attila**	**Jones**	Icahn School of Medicine at Mount Sinai	attila.g.jones@gmail.com	
**Chaggai**	**Rosenbluh**	Icahn School of Medicine at Mount Sinai	chaggai.rosenbluh@MSSM.EDU	
**Sean**	**Cho**	Kennedy Krieger Institute	sean.cho@JHMI.EDU	
**Ben**	**Langmead**	Kennedy Krieger Institute	langmea@CS.JHU.EDU	
**Jonathan**	**Pevsner**	Kennedy Krieger Institute	pevsner@KENNEDYKRIEGER.ORG	
**Jeremy**	**Thorpe**	Kennedy Krieger Institute	jthorpe6@JHMI.EDU	
**Jennifer**	**Erwin**	Lieber Institute for Brain Development	Jennifer.Erwin@libd.org	
**Andrew**	**Jaffe**	Lieber Institute for Brain Development	Andrew.Jaffe@LIBD.ORG	
**Michael**	**McConnell**	Lieber Institute for Brain Development	mikemc@libd.org	
**Rujuta**	**Narurkar**	Lieber Institute for Brain Development	Rujuta.Narurkar@LIBD.ORG	
**Apua**	**Paquola**	Lieber Institute for Brain Development	apuapaquola@GMAIL.COM	
**Jooheon**	**Shin**	Lieber Institute for Brain Development	jooheon.shin@LIBD.ORG	
**Richard**	**Straub**	Lieber Institute for Brain Development	Richard.Straub@LIBD.ORG	
**Daniel**	**Weinberger**	Lieber Institute for Brain Development	drweinberger@LIBD.ORG	
**Alexej**	**Abyzov**	Mayo Clinic Rochester	Abyzov.Alexej@MAYO.EDU	
**Taejeong**	**Bae**	Mayo Clinic Rochester	Bae.Taejeong@MAYO.EDU	
**Yeongjun**	**Jang**	Mayo Clinic Rochester	Jang.Yeongjun@mayo.edu	
**Cindy**	**Molitor**	Sage Bionetworks	cindy.molitor@SAGEBASE.ORG	
**Mette**	**Peters**	Sage Bionetworks	mette.peters@SAGEBASE.ORG	
**Fred**	**Gage**	Salk Institute for Biological Studies	gage@SALK.EDU	
**Sara**	**Linker**	Salk Institute for Biological Studies	slinker@SALK.EDU	
**Patrick**	**Reed**	Salk Institute for Biological Studies	preed@SALK.EDU	
**Meiyan**	**Wang**	Salk Institute for Biological Studies	mwang@SALK.EDU	
**Alexander**	**Urban**	Stanford University	aeurban@STANFORD.EDU	
**Bo**	**Zhou**	Stanford University	bo.zhou@STANFORD.EDU	
**Xiaowei**	**Zhu**	Stanford University	xwzhu@STANFORD.EDU	
**Reenal**	**Pattni**	Stanford University	reenal@stanford.edu	

**Aitor**	**Serres Amero**	Universitat Pompeu Fabra	aitor.serres@UPF.EDU	
**David**	**Juan**	Universitat Pompeu Fabra	david.juan@UPF.EDU	
**Irene**	**Lobon**	Universitat Pompeu Fabra	irenelobong@GMAIL.COM	
**Tomas**	**Marques-Bonet**	Universitat Pompeu Fabra	tomas.marques@UPF.EDU	
**Manuel**	**Solis Moruno**	Universitat Pompeu Fabra	manuelsolismoruno@GMAIL.COM	
**Raquel**	**Garcia Perez**	Universitat Pompeu Fabra	raquel.garcia@UPF.EDU	
**Inna**	**Povolotskaya**	Universitat Pompeu Fabra	inna.povolotskaya@UPF.EDU	
**Eduardo**	**Soriano**	University of Barcelona	esoriano@ub.edu	
**Gary**	**Mathern**	University of California, Los Angeles	gmathern@UCLA.EDU	
**Danny**	**Antaki**	University of California, San Diego	dantakli@UCSD.EDU	
**Dan**	**Averbuj**	University of California, San Diego	dan.averbuj@GMAIL.COM	
**Laurel**	**Ball**	University of California, San Diego	llball@UCSD.EDU	
**Martin**	**Breuss**	University of California, San Diego	mbreuss@UCSD.EDU	
**Eric**	**Courchesne**	University of California, San Diego	ecourchesne1949@GMAIL.COM	
**Joseph**	**Gleeson**	University of California, San Diego	jogleeson@ucsd.edu	
**Xiaoxu**	**Yang**	University of California, San Diego	xiy010@health.ucsd.edu	
**Changuk**	**Chung**	University of California, San Diego	chchung@health.ucsd.edu	
**Sarah**	**Emery**	University of Michigan	sbherman@MED.UMICH.EDU	
**Diane**	**Flasch**	University of Michigan	daflasch@UMICH.EDU	
**Jeffrey**	**Kidd**	University of Michigan	jmkidd@MED.UMICH.EDU	
**Huira**	**Kopera**	University of Michigan	chongh@UMICH.EDU	
**Kenneth**	**Kwan**	University of Michigan	kykwan@UMICH.EDU	
**Ryan**	**Mills**	University of Michigan	remills@UMICH.EDU	
**John**	**Moldovan**	University of Michigan	jmoldova@UMICH.EDU	
**John**	**Moran**	University of Michigan	moranj@UMICH.EDU	
**Chen**	**Sun**	University of Michigan	cnsun@umich.edu	
**Xuefang**	**Zhao**	University of Michigan	xuefzhao@UMICH.EDU	
**Weichen**	**Zhou**	University of Michigan	arthurz@UMICH.EDU	
**Frisbie**	**Trenton**	University of Michigan	friz@umich.edu	
**Yifan**	**Wang**	University of Michigan / Mayo Clinic Rochester	Wang.Yifan@mayo.edu	
**Adriana**	**Cherskov**	Yale University	adriana.cherskov@YALE.EDU	
**Liana**	**Fasching**	Yale University	liana.fasching@YALE.EDU	
**Alexandre**	**Jourdon**	Yale University	alexandre.jourdon@YALE.EDU	
**Sirisha**	**Pochareddy**	Yale University	sirisha.pochareddy@YALE.EDU	
**Soraya**	**Scuderi**	Yale University	soraya.scuderi@YALE.EDU	
**Nenad**	**Sestan**	Yale University	nenad.sestan@YALE.EDU	
**Flora**	**Vaccarino**	Yale University	flora.vaccarino@YALE.EDU	
