# Supplementary material for: Comprehensive identification of somatic nucleotide variants in human brain tissue
Source: Genome Biol. 2021 Mar 29;22:92. doi: 10.1186/s13059-021-02285-3 (PMC8006362; doi:10.1186/s13059-021-02285-3)
Supplement: Supplementary file 6 — Additional file 6. Review history. [file 13059_2021_2285_MOESM6_ESM.docx]

Review History

**First round of review**

**Reviewer 1**

**Are you able to assess all statistics in the manuscript, including the appropriateness of statistical tests used?** No

**Comments to author:**

The authors present a thorough evaluation of the methods to detect somatic SNVs in the brain, using whole-genome sequencing of DNA mixtures and actual samples in order to identify best practices, without requiring matched 'normal' samples. Such guidelines are very valuable, as most work on somatic variants has been done in the context of cancer, and likely differences exist with the brain and other tissues in the absence of a proper control sample.

Please find my minor questions below.

Sincerely,

Wouter De Coster

1) The provided pipeline is very helpful, just like the PON mask. While I could not find an explicit mention of this, I have the impression that this PON is only available for GRCh37?

2) The results using standard tools like Mutect2 and Strelka are sobering. I wonder if the authors have an opinion on this disappointing performance and if this can be attributed to these tools being mostly developed for tumor-normal based variant calling in a cancer setting, or are there other systematic issues?

3) While some of the contributing groups also performed WES the paper mainly discusses WGS data analysis. Are there specific recommendations for using WES? One difference that comes to mind is the more uneven coverage distribution owing to PCR amplification.

4) Do the authors think using UMIs could have an impact?

**Reviewer 2**

**Are you able to assess all statistics in the manuscript, including the appropriateness of statistical tests used?** No

**Comments to author:**

This is a technical tour de force effort by a cluster of investigators in the Brain Somatic Mosaicism Network, aiming to develop a systematic strategy to call somatic mutations from deep whole genome sequencing data on non-cancerous tissues, and to provide a best practice to the community. To this end, the authors followed a carefully designed plan, with many experimental and computational components being carried out by multiple groups in BSMN independently, followed by a joint analysis effort. The experimental part is quite comprehensive and rigor. They generated sequencing data from cell line mixtures with known variant frequencies to identify the most appropriate combination of variant calling methods and various parameters. Multiple groups in BSMN also performed independent deep sequencing (WGS, and some single-cell whole genome sequencing) on different tissue/cell types of the same donor for the calling of somatic mutations. Candidate variants were further validated extensively, with 10X linked reads and digital PCR. These data really represent what is possible nowadays with the cutting edge technologies. The computational component was very systematic. The biggest challenge with somatic variant calling is dealing with false positives. The authors carefully examined four different sources of false positives, and came up with strategies to filter them out. Some informative findings include how CNVs lead to false positives and how effective haplotype-based error detection is using 10X linked reads. The best practice developed in this study is to my knowledge the most comprehensive guideline for calling somatic SNVs, and is supported by a highly rigorous experimental validation. The authors made their data, and the entire computational workflow available at bsmn.synapse.org as well as github.com/bsmn/bsmn-pipeline, which greatly facilitates the adoption by the community. Overall, I believe this paper would be of great interests to investigators in the field of genetics, medical genetics, neuroscience and even cancer genetics.

I have only one critique. The authors stated that with their best practice they achieved a sensitivity and specificity of 90% on mosaic variant calling. This is probably the best estimate based on the results they obtained in this study. Nonetheless, most likely the true sensitivity and specificity depends on the variant frequency: variants at lower frequencies are always more difficult to call. The sensitivity and specificity of 90% was calculated based on the 43 variants and their allele frequencies present in that particular donor. If they were applying this to other donors, specificity might stay more or less the same, but sensitivity can fluctuate a lot depending on the spectrum of variant frequencies. With only 43 variants called in this study, I don't think it is feasible to report the sensitivity at different ranges of variant frequencies. Nonetheless, I would strongly recommend that the authors discuss how to interpret these performance metrics.

**Authors Response**

**Point-by-point responses to the reviewers’ comments:**

**Reviewer 1**

The authors present a thorough evaluation of the methods to detect somatic SNVs in the brain, using whole-genome sequencing of DNA mixtures and actual samples in order to identify best practices, without requiring matched 'normal' samples. Such guidelines are very valuable, as most work on somatic variants has been done in the context of cancer, and likely differences exist with the brain and other tissues in the absence of a proper control sample.

**Response:** We thank this reviewer for their positive comments and thorough evaluation of our manuscript.

1) The provided pipeline is very helpful, just like the PON mask. While I could not find an explicit mention of this, I have the impression that this PON is only available for GRCh37?

**Response:** We thank the reviewer for asking for clarification of this important point. Actually, because the high-coverage data in the 1000 Genomes Project samples is mapped to GRCh38, the PON mask used in our study is only available for GRCh38. We used a UCSC liftOver tool to map candidate somatic variants to GRCh38 and then applied the PON filter. We now explicitly state the reference genome for PON mask in the ***“Data availability”*** section of the revised manuscript.

**Excerpts from the manuscript:** The PON mask for GRCh38 can be accessed from: <https://www.synapse.org/#!Synapse:syn22024464>. The PON mask was generated using 1000 Genomes Project high

coverage	whole	genome	sequencing	of	2504	individuals	available	at	[ftp://ftp-](ftps://ftp-)
[trace.ncbi.nlm.nih.gov/1000genomes/ftp/1000G_2504_high_coverage.](http://trace.ncbi.nlm.nih.gov/1000genomes/ftp/1000G_2504_high_coverage.)

2) The results using standard tools like Mutect2 and Strelka are sobering. I wonder if the authors have an opinion on this disappointing performance and if this can be attributed to these tools being mostly developed for tumor-normal based variant calling in a cancer setting, or are there other systematic issues?

**Response:** We thank the reviewer for asking for clarification and welcome the opportunity to clarify this point in our revised manuscript. Briefly, because the Mutect2 and Strelka callers were developed to detect variants in a tumor/normal-based cancer setting, we believe these tools lack the power to detect somatic variants present (even at different frequencies) in each of the compared samples. To address this point, we have expanded the manuscript section entitled, **“*The detection of simulated somatic SNVs in DNA mixing experiments***”.

**Excerpts from the manuscript:** Moreover, MuTect2 and Strelka2, which are designed to detect somatic SNVs that are present in tumors but not matched normal samples, lacked the sensitivity to detect simulated mosaic SNVs shared between two matched samples (**Figs. 1b & Additional file 1: Fig. S2**). Indeed, given the mixing proportion and sequencing coverage, the p-value of the difference in VAFs between Mix 1 and Mix 2 for a simulated somatic SNV is around 0.01. Thus, because there are ~4 million SNPs in an individual genome, the above p-value is not sufficient to differentiate somatic SNVs from germline SNPs.

3) While some of the contributing groups also performed WES the paper mainly discusses WGS data analysis. Are there specific recommendations for using WES? One difference that comes to mind is the more uneven coverage distribution owing to PCR amplification.

**Response:** We thank this reviewer for asking this question. Briefly, our workflow can be applied for WES libraries. However, due to uneven coverage of sequence data across the genome, the detection sensitivity of mosaic variants can vary in WES data. Moreover, filtering out germline variants in duplicated genomic regions is difficult due to the lack of precise copy number estimates from WES data; thus, we mainly focused our analyses on WGS data. In the revised manuscript, we now have added a relevant text to the ***“Discussion”*** section to explicitly address this point.

**Excerpts from the manuscript:** In principle, the same workflow described above also can be applied to WES data. However, due to uneven coverage of sequence data across the genome, the detection sensitivity of mosaic variants can vary in WES data. Moreover, filtering out germline variants in duplicated genomic regions is difficult due to the lack of precise copy number estimates from WES data; thus, we mainly focused our analyses on WGS data.

4) Do the authors think using UMIs could have an impact?

**Response:** Yes, we believed that using UMIs in conjunction with duplex sequencing may improve mosaic variant calling accuracy. However, the use of UMIs for calling mosaic variants will likely require significantly greater sequencing depth; thus, the genome-wide application of using UMIs in conjunction with duplex sequencing is currently impractical. We now discuss this point in the ***“Introduction”*** section of our revised manuscript.

**Excerpts from the manuscript:** Similarly, molecular barcoding approaches such as duplex sequencing can correct errors introduced by PCR amplification or sequencing and offer >10,000-fold improvement of accuracy compared to conventional WGS. However, the most accurate molecular consensus approaches require extremely high sequencing depth (1000X or higher) to ensure that each DNA molecule is sequenced multiple times thereby effectively utilizing only a few percent of generated reads for variant calling. This requirement practically restricts the main benefit of barcoding to targeted approaches.

**Reviewer 2**

This is a technical tour de force effort by a cluster of investigators in the Brain Somatic Mosaicism Network, aiming to develop a systematic strategy to call somatic mutations from deep whole genome sequencing data on non-cancerous tissues, and to provide a best practice to the community. To this end, the authors followed a carefully designed plan, with many experimental and computational components being carried out by multiple groups in BSMN independently, followed by a joint analysis effort. The experimental part is quite comprehensive and rigor. They generated sequencing data from cell line mixtures with known variant frequencies to identify the most appropriate combination of variant calling methods and various parameters. Multiple groups in BSMN also performed independent deep sequencing (WGS, and some single-cell whole genome sequencing) on different tissue/cell types of the same donor for the calling of somatic mutations. Candidate variants were further validated extensively, with 10X linked reads and digital PCR. These data really represent what is possible nowadays with the cutting edge technologies. The computational component was very systematic. The biggest challenge with somatic variant calling is dealing with false positives. The authors carefully examined four different sources of false positives, and came up with strategies to filter them out. Some informative findings include how CNVs lead to false positives and how effective haplotype-based error detection is using 10X linked reads. The best practice developed in this study is to my knowledge the most comprehensive guideline for calling somatic SNVs, and is supported by a highly rigorous experimental validation. The authors made their data, and the entire computational workflow available at [bsmn.synapse.org](http://bsmn.synapse.org) as well as [github.com/bsmn/bsmn-pipeline,](http://github.com/bsmn/bsmn-pipeline,) which greatly facilitates the adoption by the community. Overall, I believe this paper would be of great interests to investigators in the field of genetics, medical genetics, neuroscience and even cancer genetics.

**Response:** We thank this reviewer for their kind words and thorough evaluation of our manuscript.

I have only one critique. The authors stated that with their best practice they achieved a sensitivity and specificity of 90% on mosaic variant calling. This is probably the best estimate based on the results they obtained in this study. Nonetheless, most likely the true sensitivity and specificity depends on the variant frequency: variants at lower frequencies are always more difficult to call. The sensitivity and specificity of 90% was calculated based on the 43 variants and their allele frequencies present in that particular donor. If they were applying this to other donors, specificity might stay more or less the same, but sensitivity can fluctuate a lot depending on the spectrum of variant frequencies. With only 43 variants called in this study, I don't think it is feasible to report the sensitivity at different ranges of variant frequencies. Nonetheless, I would strongly recommend that the authors discuss how to interpret these performance metrics.

**Response:** We thank the reviewer for raising this important question. We believe our sensitivity estimate is precise but agree that this point was not adequately described in our original manuscript. Briefly, in addition to estimating sensitivity using the 43 validated mosaic SNVs, we also estimated sensitivity using simulated variants from the DNA mixing experiments (see Figure 4b & Figure S10). In the revised manuscript, we added Figure S10 to clarify this important point. Figure S10 demonstrates that the estimated sensitivity across the whole genome is ~65% when considering mosaic SNVs with ~0.02 to ~0.25 VAFs. Figure 4b demonstrates that the estimated sensitivity is ~90% when considering the accessible genome (P-bases).

**Excerpts from the manuscript:** The sensitivity to detect simulated SNVs across the entire genome at VAFs ranging from -0.02 to -0.25 was -65% (**Fig. 4b**) but increased to -90% when we only considered simulated SNVs present in the accessible portion of the genome (**Additional file 1: Fig. S10**). The latter estimate is comparable to what we observed for a subset of 35
